# Palmo-plantar hyperkeratosis associated with HTLV-1 infection: a case report

**DOI:** 10.1186/s12879-021-06334-x

**Published:** 2021-07-06

**Authors:** Elías Quintero-Muñoz, Daniel Martin Arsanios, María Fernanda Estupiñán Beltrán, Juan David Vera, Catalina Palacio Giraldo, Omar Velandia, Carlos Mauricio Calderon

**Affiliations:** 1 General Medicine, La Sabana University, Chía, Colombia; 2 Internal Medicine resident, La Sabana University, Chía, Colombia; 3 Internal Medicine, La Samaritana Hospital, Bogotá, Colombia

**Keywords:** HTLV, Palmoplantar keratoderma, *Strongyloides stercoralis*, Malabsortive diarrhea, Case report

## Abstract

**Background:**

Palmoplantar hyperkeratosis is a cutaneous manifestation that had not been clearly associated with infection by the human T-cell lymphotropic virus, which is a retrovirus that in most cases does not develop clinical pathologies and its symptoms may be undetected. The skin is one of the most affected organs, however until now only seborrheic dermatitis, xerosis/ichthyosis and infective dermatitis associated with HTLV-1 have been described as cutaneous clinical manifestations of this disease.

**Case presentation:**

We present the case of a 36-year-old male patient with serologically documented HTLV-1 infection, who presented symptoms of diarrhea, malabsorption due to *Strongyloides stercoralis*, and in whom a physical examination revealed an association with generalized xerosis and palmoplantar keratoderma confirmed by skin biopsy. Other infectious etiologies and malignancy were ruled out. This clinical manifestation was managed with dermal hydration, and skin care which improved the thickened skin and make it less noticeable.

**Conclusions:**

According to our experience, this is the first reported case of palmoplantar keratoderma associated with a human lymphotropic virus infection. This is a skin manifestation that has not been confirmed in conjunction with HTLV-I before. This implies that palmoplantar keratoderma is a new clinical manifestation of this infection, that should be considered in the initial approach of patients in endemic areas with these dermatological characteristics.

## Background

Human T-cell Lymphotropic Virus (HTLV) is a retrovirus first isolated in 1980 from a T lymphocyte in a patient with cutaneous lymphoma (HTLV-1), after that, HTLV-2 was identified in cells from a patient with hairy cell leukemia [1, 2]. Despite the fact that most HTLV-1 infections are usually asymptomatic, in some cases it has been associated with multiple pathologies that produce high morbidity and mortality, such as Adult T-cell Leukemia/Lymphoma (ATLL), Myelopathy / Tropical Spastic Paraparesis (TSP) and a wide spectrum of skin involvement, the skin being one of the organs most affected by this virus [3–6]. HTLV-2 infection, has not been consistently related to any pathology, there are few case reports that relate it to neurological diseases [7].

HTLV-1 infection is endemic in southern Japan, the Caribbean, Central and South America, some regions of Africa, also Australia and the Pacific [8]. The development of clinical pathologies is rare and only 3–5% of carriers manifest them; this situation depends on the age, time of infection and immune system state [9]. The wide range of manifestations may be due to virus-dependent cellular transformation or alterations generated on the immune status of the host [10].

Palmoplantar keratodermas (PPK) are a heterogeneous group of keratinization disorders with hyperkeratotic thickening of palms and soles. The etiology can be hereditary, caused by mutations in genes that encode proteins that are components of the intracellular cytoskeleton such as keratin or that participate in intercellular adhesion, cell-to-cell communication (for example, Connexins) and cell signaling. The other group of PPK are acquiredes which are secondary to toxic, infectious diseases and malignant neoplasms including esophageal cancer, Hodgkin’s disease, prolymphocytic leukemia, renal, mammary, pancreatic, colonic adenocarcinomas and malignant melanoma [11].

We present the case of a patient with serologically documented HTLV infection associated with acquired palmoplantar keratoderma transgrediens, a cutaneous manifestation of HTLV not clearly described in the literature, also in the context of an adult patient with malabsorptive diarrhea and *Strongyloides stercoralis* infection.

## Case presentation

**Fig. 1:**
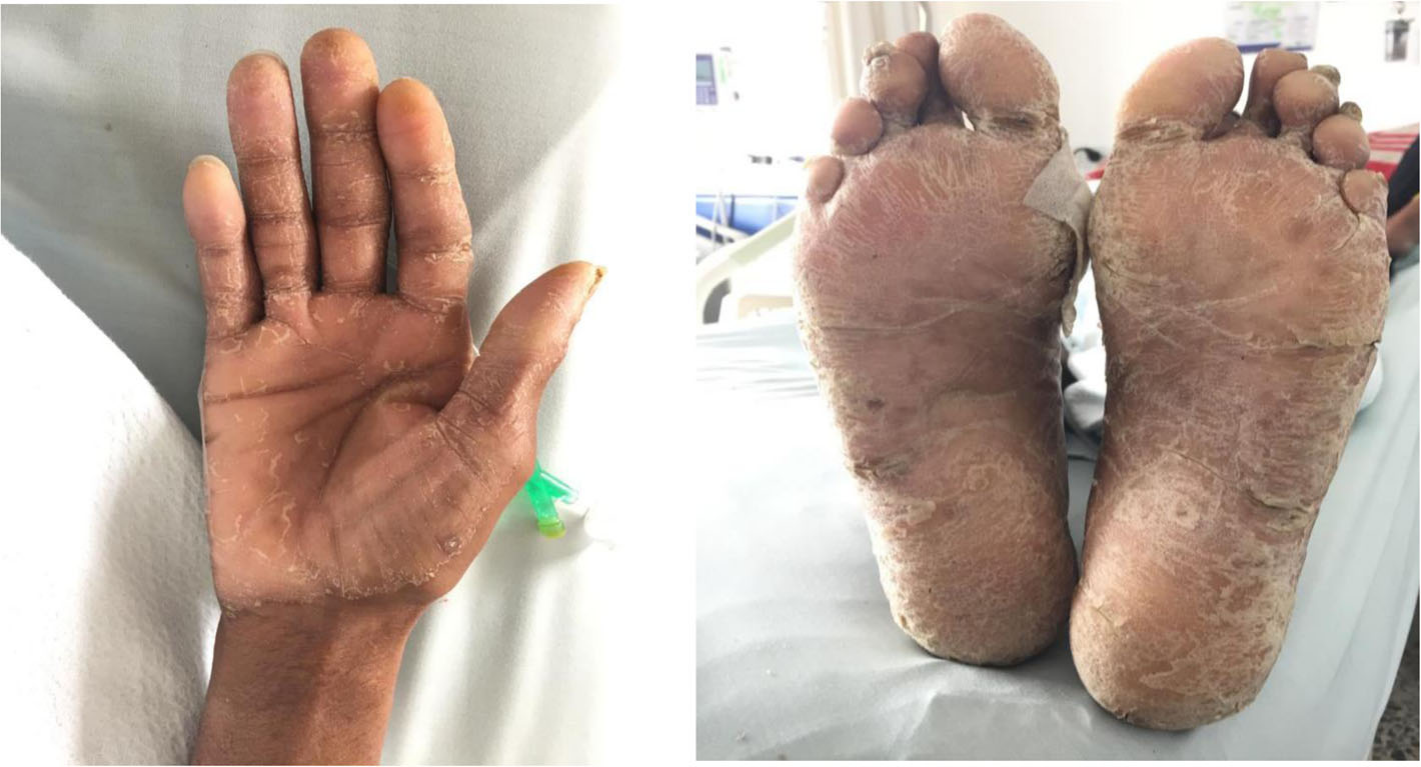
Palmoplantar hyperkeratosic lesions and xerosis. (a) Palms (b) plant

A 36-year-old male patient, from Valle del Cauca, Colombia, incarcerated in a penitentiary center, without medical or family history, who was admitted to the emergency department due to a 3-month clinical course consisting of liquid stool, approximately 7 episodes daily, associated with multiple emetic episodes of food content and weight loss. In addition, patient reported an 8-month history of palmoplantar hyperkeratosis with onychrygriphosis and generalized cutaneous xerosis (Fig. 1), without treatment. Apart from skin lesions, no other relevant findings of the physical examination were found.

Among the laboratory studies carried out, the presence of positive serology for HTLV-1 and other negative infectious studies stands out. To rule out malignancy, bone marrow studies were performed, including biopsy, which had no evidence of malignancy (Table 1). A total abdominal ultrasound was also performed, which ruled out the presence of abdominal masses including renal masses. A colonoscopy was performed that only reported external hemorrhoids without mass findings. Diarrheal disease with malabsorption characteristics was considered and rifaximin treatment was started (200 mg three times a day for 3 days), however, it did not have an adequate response to treatment; Subsequently, an endoscopy of the upper digestive tract was performed with a finding of erosive bulbooduodenitis with a biopsy that revealed abundant forms of *Strongyloides stercoralis*, so treatment with ivermectin was started (200 mcg/kg/dose), with the consequent decrease in the frequency and volume of bowel movements.

**Table 1:** Laboratory results

Blood count		Infectious profile		Coproscopic	Bone marrow studies
Leukocytes (cell/mm^3^)	10,170	HIV	Negative	No parasitic structures are observed in the analyzed sample	**Peripheral blood smear:** Normal white blood cells in number and morphology neutrophils: 69% lymphocytes: 20% monocytes: 10% Eosinophils: 1%. Red blood cell morphology with mild anisocytosis and hypochromia. Increased platelet count and occasional macro platelets. **Conclusion:** Morphological abnormalities in lymphocytes with a cleaved nucleus, associated with neutrophilia with evidence of Pelger Hüet abnormality and significant thrombocytosis
Neutrophils (cell/mm^3^)	5340	Non-treponemal test	No reactive		
Lymphocytes (cell/mm^3^)	3620	HBsAg	Negative		
Monocytes (cell/mm^3^)	1110	HTLV serology	80.33 (Positive)		**Bone marrow biopsy:** Hypocellular bone marrow for age with alteration in the myeloid and megakaryocytic lines.
Eosinophils (cell/mm^3^)	104				
Haemoglobin (g/dl)	13				
Haematocrito (%)	38.5				
Platelets (cell/mm^3^)	559,000				

**Fig. 2:**
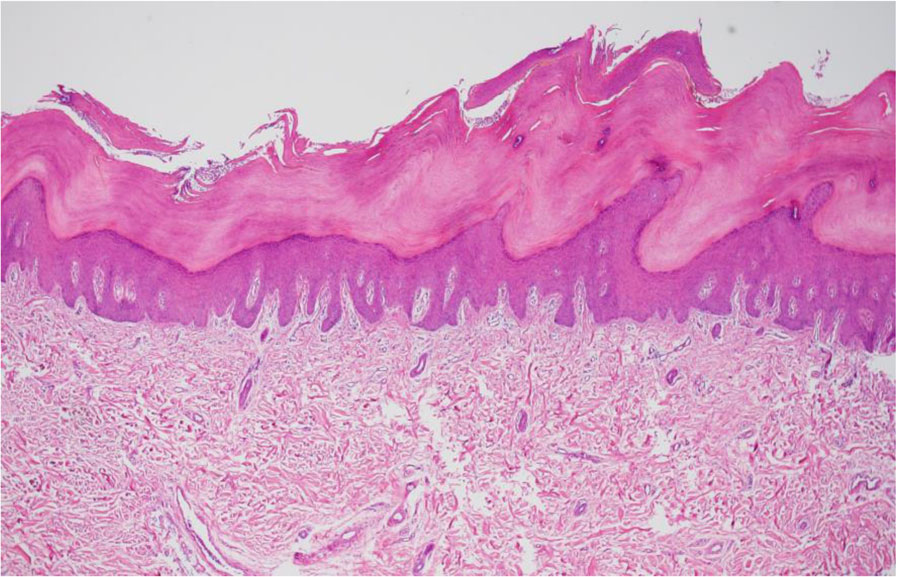
Skin biopsy

It was decided to manage with dermal hydration and skin care, including emollients, as part of the treatment of PPK, which was confirmed by skin biopsy (Fig. 2). The PPK was thought to be related to HTLV-1, due to the wide spectrum of cutaneous manifestation that the infection can cause, although, this association has not been previously reported.

## Discussion/conclusion

Although HTLV-1 infection in most cases does not generate disease, it has been described that many of these patients are not entirely asymptomatic and are compromised undetected, the skin is one of the most affected organs, as evidenced by the series of cases in the blood donors [6, 12]. This fact becomes more important since our territory is recognized as an endemic area, with epidemiological data corresponding to a prevalence between 0.17–0.3%, the most affected regions are the Pacific, Caribbean and Andean region [13, 14].

Among the characteristic cutaneous manifestations directly related to infection by HTLV-1 are infectious dermatitis, crusted scabies, and skin injuries by ATLL and MPET [12, 15, 16]. According to a series of cases with more patients, in a population of Brazil, they evidenced a prevalence of skin alteration in asymptomatic seropositive patients of 76% and in those with MPET it was 88%. Among the findings, inflammatory pathologies predominated, acquired xerosis/ichthyosis and seborrheic dermatitis were the most frequent skin disorders in the adult population, with a prevalence of 60.2/42.5% (xerosis / ichthyosis) and 47.9/15% (seborrheic dermatitis) for the asymptomatic and MPET groups respectively; likewise, infectious diseases obtained a prevalence of 30%, with dermatophytosis and scabies being the most prevalent [17]. On the other hand, infectious dermatitis prevails in the child population, who are characterized by presenting generalized eczema and *Staphylococcus* and *Streptococcus* infections who require chronic antibiotic treatment because of high recurrence rate [4, 5, 18]. In 1993, Nagatani, et al. published a case report showing a possible association between PPK and HTLV-1, but a definitive diagnosis was not confirmed by biopsy [19].

Our case is relevant because it presents a cutaneous manifestation, confirmed by biopsy, which has not been described in association with HTLV-1 infection, it presents generalized cutaneous xerosis associated with PPK transgrediens, which is a condition consisting of the abnormal thickening of the epidermis of palms and plants, and additionally has the ability to affect areas beyond the palmoplantar skin, these qualities differentiate it from acquired ichthyosis, since it does not meet the clinical characteristics of the adherent visible scales, nor the alteration in the granular layer, which are documented structural characteristics in the biopsy; therefore, this pathology is ruled out.

PPK is considered an independent clinical entity that is part of the palmoplantar keratoderma group and the approach of the etiology requires the assessment of multiple systemic and non-systemic conditions that may be associated with its presentation, including infectious, malignancy, endocrine and autoimmune diseases, among others. PPK has been associated with perimenopause, the use of drugs, or infectious processes such as dermatophytosis, human papilloma virus, syphilis, leprosy, miliary tuberculosis, and scabies (ruled out pathologies in the case report) but never with HTLV [20]. Particularly, PPK secondary to human papilloma virus is especially presented in immunocompromised patients and the usual presentation is confluent verrucous masses on the palms and soles. Our case is about a patient without laboratory alterations suggesting compromise in the immune system, similarly, skin demonstrations were not the usual reported for this infection. Our experience suggests that PPK is a newly recognised cutaneous manifestation associated with HTLV-1 infection.

Furthermore, the association between HTLV-1 infection and *Strongyloides stercolaris* is known. The available evidence suggest HTLV-1 infection is more frequently associated (up to 2.4 times more) with *Strongyloides stercolaris* infection [21, 22]. Similarly, it has been suggested that Strongyloidiasis increases the risk of diseases associated with HTLV-1, modifying its viral load [23], a view that this coinfection is considered a possible risk factor for the development of lymphoproliferative syndromes associated with this virus, or even serve as a marker of severity of HTLV-1 infection [22]. *Strongyloides* hyperinfection syndrome, is a severe form of strongyloidiasis, has been described in patients with drug immunosuppression or secondary to malignancy, but also in cases of HTLV-1 infection [24]. Although strongyloidiasis has been associated with malabsorption syndromes, there is no clear association in literature suggesting a relationship between PPK and malabsorption syndromes of another etiology. This case is about a patient who had no clinical or paraclinical demonstration suggesting any particular systemic condition.

According to that, the evaluation of this coinfection and this cutaneous manifestation not previously described in relation to HTLV-1 infection, is important in order to carry out an adequate evaluation and follow-up, monitoring the possible progression of the disease and the development of complications to short and long term in an endemic region.

## Data Availability

All data and materials of this article are included in the manuscript.

## Abbreviations

HTLV: Human T-cell Lymphotropic Virus
ATLL: Adult T-cell Leukemia/Lymphoma
TSP: Myelopathy / tropical spastic paraparesis
PPK: Palmoplantar Keratoderma
